# *Isotrema sanyaense*, a new species of Aristolochiaceae from Hainan, China

**DOI:** 10.3897/phytokeys.128.35042

**Published:** 2019-08-12

**Authors:** Rongtao Li, Zhiwei Wang, Jun Wang, Xinxin Zhu, Han Xu

**Affiliations:** 1 Hainan Branch of the Institute of Medicinal Plant Development, Chinese Academy of Medical Sciences & Peking Union Medical College, Haikou, Hainan, 570100, China Hainan Branch of the Institute of Medicinal Plant Development, Chinese Academy of Medical Sciences Haikou China; 2 College of Pharmacy, Guizhou University of Traditional Chinese Medicine, Guiyang, Guizhou, 550002, China Guizhou University Guiyang China; 3 College of Life Sciences, Xinyang Normal University, Xinyang, Henan, 464000, China Xinyang Normal University Xinyang China; 4 Research Institute of Tropical Forestry, Chinese Academy of Forestry, Longdong, Guangzhou 510520, China Research Institute of Tropical Forestry, Chinese Academy of Forestry Guangzhou China

**Keywords:** *
Aristolochia
*, Aristolochia subgenus Siphisia, Asia, morphology, taxonomy

## Abstract

*Isotrema sanyaense* R.T.Li, X.X.Zhu & Z.W.Wang, **sp. nov.**, a new species from Hainan island, China, is described and illustrated here. It is morphologically most similar to *I. ledongense* (Han Xu, Y.D.Li & H.J.Yang) X.X.Zhu, S.Liao & J.S.Ma and *I. jianfenglingense* (Han Xu, Y.D.Li & H.Q.Chen) X.X.Zhu, S.Liao & J.S.Ma in the shape of leaf, flower, and the yellow to brown villous indumentum of the pedicel, ovary and calyx. However, *I. sanyaense* can be easily distinguished from the latter two species by its 1–5-flowered cymes, in hanging clusters of 1 to numerous branches, upper calyx tube obviously longer than basal calyx tube, calyx limb discoid, yellow inside, with purple-red stripes and spots, about 13–18 mm in diameter, glabrous, and a throat dark red without spots, 4–6 mm wide.

## Introduction

*Isotrema* Raf. (Aristolochiaceae), previously treated as a subgenus of *Aristolochia* L., was recently reinstated as an independent genus based on molecular and morphological evidence (Zhu et al. 2019). It differs from other genera of Aristolochiaceae by a combination of characters: calyx strongly curved, gynostemium 3-lobed, anthers paired on the outer surface of each gynostemium segment, and capsule dehiscing basipetally (Do et al. 2015a, Zhu et al. 2019). Currently, *Isotrema* comprises 99 species and one subspecies, mainly distributed in East and South Asia, with some species scattered in North and Central America (Zhou et al. 2019, Zhu et al. 2019). China accommodates 59 species and one subspecies, of which 47 species and one subspecies are endemic to the country (Hwang et al. 2003, Zhou et al. 2019, Zhu et al. 2019).

During our field investigations to South Hainan Province, China, in 2017 and 2018, an unknown species of Aristolochiaceae was discovered. The horseshoe-shaped calyx tube, 3-lobed calyx limb and gynostemium, anthers adnate in pairs opposite the gynostemium lobes, and capsule dehiscing basipetally indicate it to be a member of *Isotrema*. After comparing with other species of the genus, we confirmed that the unidentified species from Hainan island represents a new taxon, so here, we describe and illustrate it.

## Material and methods

Measurements and assessments of morphological data of the species described here were based on living plants obtained in the wild. Flowering and fruiting branches were pressed to specimens and deposited in the CSH and KUN herbaria (herbarium acronyms follow Thiers 2019). The comparison among similar species was based on extensive revision of specimens (including types) of *Isotrema* in A, BM, BR, CDBI, CSFI, CSH, E, EMA, GXMI, HAST, HENU, HHBG, HIB, HITBC, HNWP, IBK, IBSC, K, KUN, KYO, L, LBG, LE, NAS, NTUF, P, PE, PEM, SM, SNU, SYS, TAI, TI, W, WCU, WU, WUK, XYTC and YUKU herbaria, as well as related literature (Cheng et al. 1988, Ma 1989a, 1989b, Hwang et al. 2003, Ohi-Toma et al. 2006, Xu et al. 2011, Do et al. 2015a, 2015b, Ohi-Toma and Murata 2016, Zhu et al. 2016, 2017a, 2017b, 2018, Gong et al. 2018, Yang et al. 2018).

## Taxonomy

### Isotrema sanyaense

Taxon classificationPlantaePiperalesApocynaceae

R.T.Li, X.X.Zhu & Z.W.Wang sp. nov.

11361fe3-58f1-514b-9213-5455b755094b

urn:lsid:ipni.org:names:77200836-1

Figs 1 , 2 , 3

#### Diagnosis.

*Isotrema sanyaense* is most similar to *I. ledongense* (Han Xu, Y.D.Li & H.J.Yang) X.X.Zhu, S.Liao & J.S.Ma and *I. jianfenglingense* (Han Xu, Y.D.Li & H.Q.Chen) X.X.Zhu, S.Liao & J.S.Ma (Zhu et al. 2019), but significantly differs in the following characters: cymes 1–5-flowered, in hanging clusters of 1 to numerous branches, the pedicel nearly equal in length to flower, upper calyx tube obviously longer than basal calyx tube, calyx limb discoid, yellow inside, with purple-red stripes and spots, about 13–18 mm in diameter, glabrous, the throat dark red without spots, 4–6 mm wide. A detailed morphological comparison among the three species is shown in Figure 4 and Table 1.

**Figure 1.**
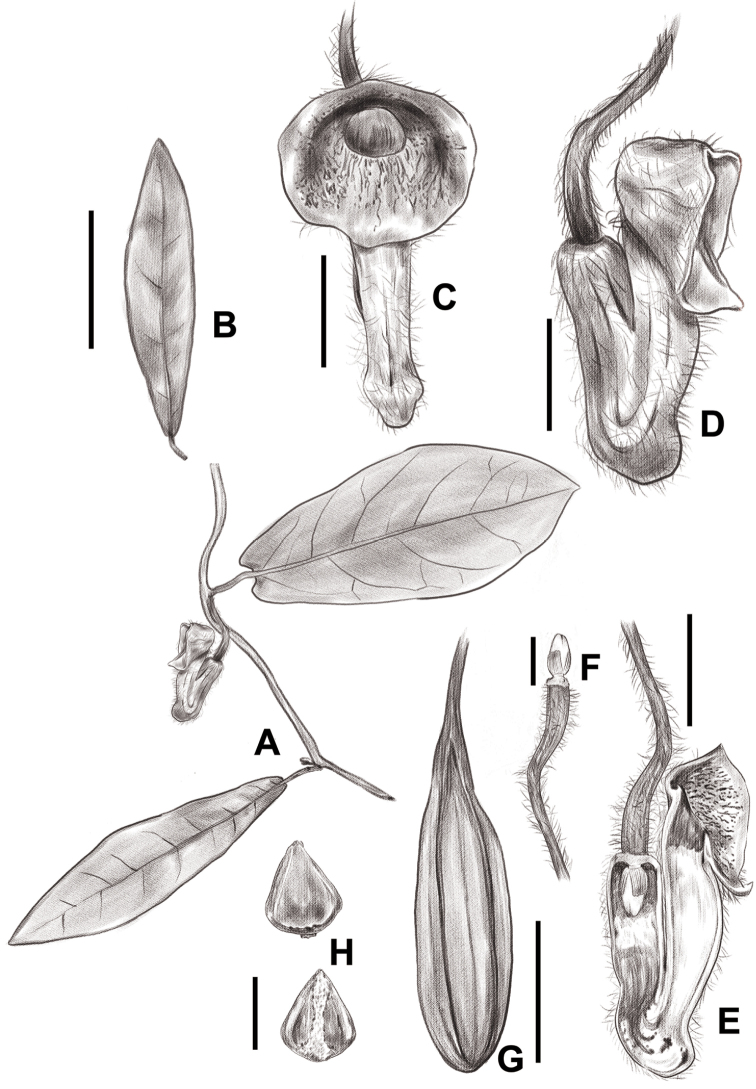
*Isotrema sanyaense* R.T.Li, X.X.Zhu & Z.W.Wang, sp. nov. **A** flowering branch **B** leaf **C** flower (front view) **D** flower (lateral view) **E** opened flower (showing the inside structure) **F** anthers and gynostemium **G** fruit **H** seeds. Scale bars: 6 cm (**B**); 1 mm (**C, D, E**); 5 mm (**F**); 2 cm (**G**); 3 mm (**H**).

**Figure 2.**
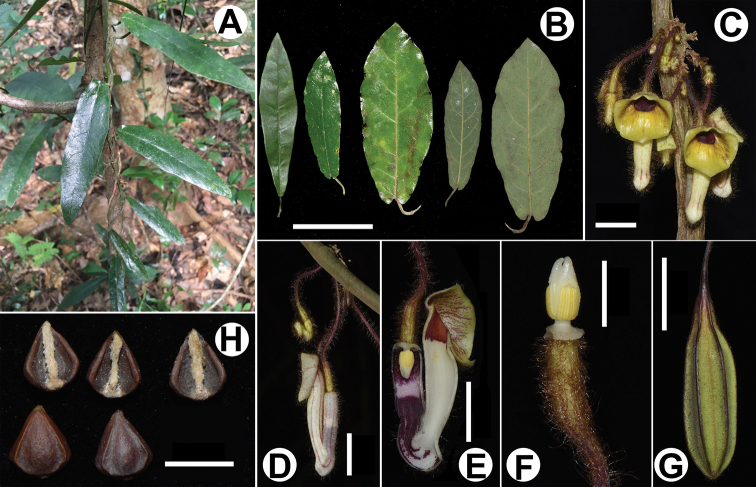
*Isotrema sanyaense* R.T.Li, X.X.Zhu & Z.W.Wang, sp. nov. **A** habit **B** leaves (adaxially and abaxially) **C** inflorescence **D** flower (lateral view) **E** opened flower (showing the inside structure) **F** anthers and gynostemium **G** fruit **H** seeds. Scale bars: 1 cm (**B, C**); 2 cm (**D, E, G**); 5 mm (**F, H**).

#### Type.

CHINA. Hainan: Sanya City, Haitang District, Haitangwan Town, 18°17'22"N, 109°39'45"E, 332m a.s.l., 28 October 2017 (fl), *X.X.Zhu & R.T.Li ZXX17105* (holotype: CSH-0146607!; isotype: CSH!, KUN!).

#### Description.

Woody liana. Young stems terete, densely villous, with yellow to brown trichomes, old branchlets glabrous, old stems leafless. Petioles 0.8–1.7 cm long, young ones densely villous, with yellow to brown trichomes mixed with a white pubescence; blades lanceolate or elliptic-lanceolate, entire, 9–14 × 3–6 cm, leathery, adaxially glabrous, abaxially villous, with sparse larger white appressed trichomes, mixed with shorter white pubescence, veins pinnate, 5 to 10 pairs, base shallowly cordate to cordate, sinus < 2–3 mm deep, apex acute. Cymes lateral on old woody stems or axillary, 1–5-flowered, in hanging clusters of 1 to numerous branches, pedicels 1.1–2.6 cm long, densely villous, with yellow to brown trichomes; bracteoles ovate-lanceolate, ca. 0.2–0.4 × 0.4 mm, inserted at the basis of pedicel, adaxially glabrous, abaxially densely villous, with yellow to brown trichomes. Calyx horseshoe-shaped, externally white with purple-red stripes; abaxially densely villous, with yellow to brown trichomes; basal tube ca. 2.2 × 0.5 cm, inside dark purple, with white patches spaced in the middle; upper tube ca. 2.5 × 0.5 cm, white inside, getting dark red in upper portion; calyx limb discoid, ca. 13–18 mm in diameter, abaxially densely villous, with yellow to brown trichomes, the inner surface yellow with purple-red stripes and spots, glabrous; throat dark red, 4–6 mm wide. Anthers 6, oblong, ca. 2.2 mm long, adnate in 3 pairs to base of gynostemium, opposite to lobes, dehiscence longitudinal. Gynostemium ca. 5 mm long, 3-lobed, apex acute; ovary inferior, 6-loculed, abaxially densely villous, with yellow to brown trichomes; ovules numerous; placentation axillary. Capsule oblong-ellipsoid, ca. 5 × 2 cm, dehiscing basipetally. Seeds triangular-ovate, 4–5 × 3–4 mm.

**Figure 3.**
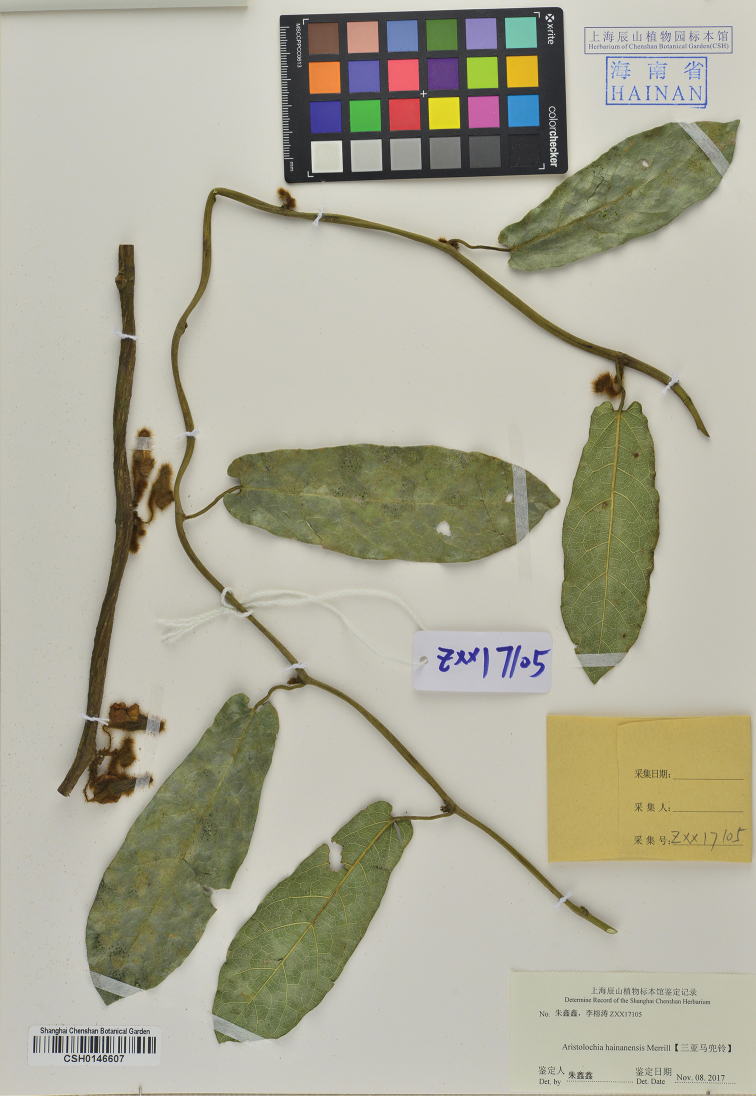
Holotype of *Isotrema sanyaense* R.T.Li, X.X.Zhu & Z.W.Wang, sp. nov. [CSH-0146607]!.

**Figure 4.**
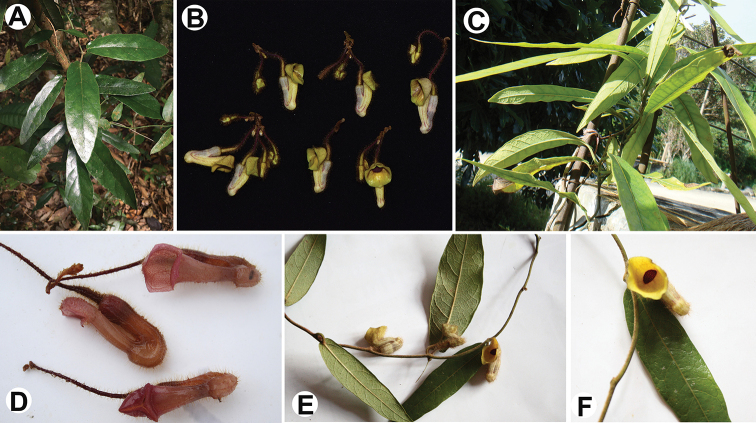
**A, B***Isotrema sanyaense* R.T.Li, X.X.Zhu & Z.W.Wang, sp. nov. **A** habit **B** flowers **C, D***I. jianfenglingense* (Han Xu, Y.D.Li & H.Q.Chen) X.X.Zhu, S.Liao & J.S.Ma **C** habit **D** flowers **E, F***I. ledongense* (Han Xu, Y.D.Li & H.J.Yang) X.X.Zhu, S.Liao & J.S.Ma **E** flowering branch **F** flower (front view).

#### Phenology.

Flowering specimens have been collected in October and in fruiting specimens in May.

#### Etymology.

The specific epithet is derived from the type locality, Sanya City, in Hainan island, China. The Chinese name is given as “三亚关木通”.

#### Distribution and habitat.

*Isotrema sanyaense* is currently known from Haitangwan Town, Haitang District, Sanya City, Hainan Province, China. It grows in lowland dry forests dominated by families including Euphorbiaceae, Fagaceae, Lauraceae, Myrtaceae, Arecaceae and Rubiaceae at elevations between 332–400 m.

#### Conservation status.

*Isotrema sanyaense* is only known from two populations in Sanya City, Hainan island, China, with fewer than 30 individuals seen at each site. Therefore, the new species is assigned a preliminary status of Vulnerable (VU D2) according to IUCN Red List Criteria (IUCN 2012), indicating a population with a very restricted area of occupancy (typically less than 20 km^2^) or number of locations (typically five or fewer).

#### Additional specimens examined (paratypes).

**CHINA. Hainan**: Sanya City, Haitang District, Haitangwan Town, 18°17'24"N, 109°39'43"E, 400m a.s.l., 28 October 2017 (vegetative), *X.X.Zhu & R.T.Li ZXX17106* (CSH); same location, 18°17'29"N, 109°39'46"E, 376m a.s.l., 21 May 2018 (fr), *X.X.Zhu & J.Wang ZXX18075* (CSH, KUN).

**Table 1.** Morphological comparison of key characters among *Isotrema sanyaense*, *I. ledongense* and *I. jianfenglingense*.

Characters	*I. sanyaense*	*I. ledongense*	*I. jianfenglingense*
Leaf blade	adaxially glabrous, abaxially sparsely with white pubescence	adaxially mixed with yellow-brown villous and white pubescence, abaxially densely yellow-brown villous	adaxially sparsely yellow-brown villous and white pubescence, abaxially mixed with yellow-brown villous and white pubescence
Inflorescence	cyme 1–5-flowered, in hanging clusters of 1 to numerous branches	solitary	solitary
Pedicel	1.1–2.6 cm, nearly equal in length to flower	0.7–1 cm, significantly shorter than flower	2.7–3.5cm, nearly equal in length to flower
Calyx	white, with purple-red stripes; basal tube ca. 22 × 5 mm, upper tube significantly longer than basal tube	light yellow, with purple-red stripes; basal tube 15–16 × 4.5–5 mm, upper tube almost equal to basal tube	light red brown, without stripes; basal tube 23–26 × 5–6 mm, upper tube significantly longer than basal tube
Limb	discoid, yellow, 13–18 mm in diameter, with purple-red stripes and spots, lobes without papillae and pubescent	discoid, yellow, 5–7 mm in diameter, only with unobvious light red spots, lobes densely papillate	trumpet-shaped, pink, 8–9 mm in diameter, lobes densely papillae and white pubescent
Throat	dark red without spots, 4–6 mm wide, significantly smaller than the limb	dark red with light yellow spots, ca. 5 mm wide, significantly smaller than the limb	pink with red-brown spots, ca. 8–9 mm wide, approximately to the limb
Gynostemium	ca. 5 mm long, lobes nearly equal in length to anthers, apex acute	ca. 3 mm long, lobes significantly shorter than anthers, apex obtuse	ca. 4 mm long, lobes significantly shorter than anthers, apex curved

## Discussion

Morphologically, *Isotrema sanyaense* resembles *I. ledongense* and *I. jianfenglingense* in having similar leaf blade shape (lanceolate or elliptic-lanceolate, entire, base shallowly cordate) and the yellow to brown villous indumentum of the pedicel, ovary and calyx, but *I. sanyaense* and *I. ledongense* are significantly different in the inflorescence (cymes 1–5-flowered, in hanging clusters of 1 to numerous branches vs. solitary), the pedicel (nearly equal in length to flower vs. significantly shorter than flower), the calyx tube (upper tube obviously longer than basal tube vs. upper tube almost equal to basal tube), the calyx limb (about 13–18 mm in diameter, with purple-red stripes and spots vs. 5–7 mm in diameter, only with unobvious light red spots), and the throat (dark red without spots vs. dark red with light yellow spots). *Isotrema sanyaense* can also be easily distinguished from *I. jianfenglingense* by the inflorescence (cymes 1–5-flowered, in hanging clusters of 1 to numerous branches vs. solitary), the calyx (white with purple-red stripes vs. light red to brown without stripes), the calyx limb (discoid, yellow, with purple-red stripes and spots, lobes without papillae and pubescent vs. trumpet-shaped, pink, without stripes and spots, lobes densely papillae and pubescent), and the throat (dark red without spots, significantly smaller than the limb width vs. pink with red-brown spots, approximately to the limb width) (summarized in Table 1). Considering the discovery of this new species from Hainan island, along with the species previously described in China, Myanmar, Peninsular Malaysia, Thailand and Vietnam in recent years (González and Poncy 1999, Phuphathanaphong 2006, Hansen and Phuphathanaphong 2009, Liu and Deng 2009, Xu et al. 2011, Yao 2012, Huang et al. 2013, 2015, Wu et al. 2013, 2015, Do et al. 2014, 2015a, 2015b, 2015c, 2015d, 2017, 2019, Huong et al. 2014, Lu and Wang 2014, Ohi-Toma et al. 2014, Ravikumar et al. 2014, Zhu et al. 2015, 2016, 2017a, 2017b, 2018, Gong et al. 2018, Yang et al. 2018, Zhou et al. 2019), we predict that more and more new species of *Isotrema* will be found after extensive investigations and studies.

### Key to *Isotrema sanyaense* and closely related species (including all species of *Aristolochia* and *Isotrema* in Hainan island, China)

**Table:** 

1	Calyx tube rectilinear or slightly curved; with short stipe connected to ovary; limb ligulate; gynostemium 6-lobed; anthers 6, opposite to lobes of gynostemium; capsule dehiscing acropetally	**2**
–	Calyx tube horseshoe-shaped at middle; without short stipe connected to ovary; limb discoid or obliquely trumpet-shaped; gynostemium 3-lobed; anthers 6, adnate in pairs opposite the gynostemium lobes; capsule dehiscing basipetally	**3**
2	Leaf blade polymorphic, ovate or ovate-deltate to sagittate, usually deeply 3-lobed, smaller, 2.5–5.5 × 2–6 cm; seeds ovoid, 2.5 × 2 mm; flowering from October to November	***A. polymorpha***
–	Leaf blade ovate-cordate or oblong-ovate, entire, larger, 8–24 × 4–22 cm; seeds triangular to subcordiform, ca. 8 × 8 mm. flowering from May to August	***A. tagala***
3	Calyx limb trumpet-shaped, throat as long to the calyx limb	**4**
–	Calyx limb discoid, bell-shaped, throat significantly shorter than the calyx limb	**5**
4	Throat yellow without spot; leaf blades ovate to ovate-lanceolate, lateral veins 5 to 7 pairs	***I. hainanense***
–	Throat pink with red-brown spots; leaf blades lanceolate to elliptic-lanceolate, lateral veins 16 to 18 pairs	***I. jianfenglingense***
5	Calyx limb discoid; width of throat > 10 mm; leaf blades polymorphic, broadly oblong-oblanceolate, linear, or oblong, widest at upper half, often shallowly 2–3-lobed	***I. howii***
–	Calyx limb bell-shaped or discoid; width of throat ≤ 6mm; leaf blades uniform, widest at middle or lower half, not lobed	**6**
6	Calyx limb bell-shaped, inner surface purple black	***I. fulvicomum***
–	Calyx limb discoid, inner surface yellow, sometimes with red stripes and spots	**7**
7	Upper calyx tube almost equal to basal calyx tube; calyx limb 5–7 mm in diameter, only with unobvious light red spots; gynostemium lobes significantly shorter than anthers, with obtuse apices	***I. ledongense***
–	Upper calyx tube significantly longer than basal calyx tube; calyx limb about 13–18 mm in diameter, with purple-red stripes and spots; gynostemium lobes nearly equal in length to anthers, with acute apices	***I. sanyaense***


## Supplementary Material

XML Treatment for Isotrema sanyaense
